# Association between behavioral phenotypes and response to a physical activity intervention using gamification and social incentives: Secondary analysis of the STEP UP randomized clinical trial

**DOI:** 10.1371/journal.pone.0239288

**Published:** 2020-10-14

**Authors:** Xisui Shirley Chen, Sujatha Changolkar, Amol S. Navathe, Kristin A. Linn, Gregory Reh, Gregory Szwartz, David Steier, Sarah Godby, Mohan Balachandran, Joseph D. Harrison, Charles A. L. Rareshide, Mitesh S. Patel

**Affiliations:** 1 Department of Medicine, University of Pennyslvania Perelman School of Medicine, Philadelphia, Pennsylvania, United States of America; 2 Penn Medicine Nudge Unit, University of Pennyslvania, Philadelphia, Pennsylvania, United States of America; 3 Crescenz Veterans Affairs Medical Center, Philadelphia, Pennsylvania, United States of America; 4 Department of Biostatistics, Epidemiology and Informatics, University of Pennyslvania Perelman School of Medicine, Philadelphia, Pennsylvania, United States of America; 5 Deloitte Consulting, Philadelphia, Pennsylvania, United States of America; University of Tennessee Health Science Center, UNITED STATES

## Abstract

Participants often vary in their response to behavioral interventions, but methods to identify groups of participants that are more likely to respond are lacking. In this secondary analysis of a randomized clinical trial, we used baseline characteristics to group participants into distinct behavioral phenotypes and evaluated differential responses to a physical activity intervention. Latent class analysis was used to segment participants based on baseline participant data including demographics, validated measures of psychosocial variables, and physical activity behavior. The trial included 602 adults from 40 U.S. states with body mass index ≥25 who were randomized to control or one of three gamification interventions (supportive, collaborative, or competitive) to increase physical activity. Daily step counts were monitored using a wearable device for a 24-week intervention with 12 weeks of follow-up. The model segmented participants into three classes named for key defining traits: Class 1, extroverted and motivated; Class 2, less active and less social; Class 3, less motivated and at-risk. Adjusted regression models were used to test for differences in intervention response relative to control within each behavioral phenotype. In Class 1, only participants in the competitive arm increased their mean daily steps during the intervention (adjusted difference, 945; 95% CI, 352–1537; *P* = .002), but it was not sustained during follow-up. In Class 2, participants in all three gamification arms significantly increased their mean daily steps compared to control during the intervention (supportive arm adjusted difference 1172; 95% CI, 363–1980; *P* = .005; collaborative arm adjusted difference 1119; 95% CI, 319–1919; *P* = .006; competitive arm adjusted difference 1179; 95% CI, 400–1957; *P* = .003) and all three had sustained impact during follow-up. In Class 3, none of the interventions had a significant effect on physical activity. Three behavioral phenotypes were identified, each with a different response to the interventions. This approach could be used to better target behavioral interventions to participants that are more likely to respond to them.

## Introduction

Modifiable behavioral risk factors such as poor diet, low physical activity, and tobacco use account for 40% of premature mortality in the United States, highlighting the need for effective behavior change interventions [1, 2]. Many behavior modification strategies appear promising, but systematic reviews and meta-analyses of randomized control trials indicate significant heterogeneity in outcomes that is poorly understood [3–5]. In particular, little is known about which interventions are effective for population sub-groups. While demographic attributes have been correlated with health behaviors [6], they often do not fully explain differences in intervention effectiveness [7–9]. Similarly, comparisons within studies suggest that baseline participant traits such as age, sex, race/ethnicity and health status are not reliable predictors of participant response [10–12].

Health behavior research could be improved by including additional data that informs factors influencing behavior change in intervention trials. For example, personality traits and psychological constructs such as affective response and resilience may elucidate important differences in how individuals perceive and engage with behavioral interventions that sociodemographic and clinical variables do not [12–14]. Evidence suggests that higher order combinations of demographic, health status, and psychosocial variables better capture the complexity of behavior change compared to any single variable type alone [15]. An analytical approach similar to audience segmentation in marketing can be applied to identify patterns of sample characteristics that describe baseline variation among participants [16]. In healthcare, segmentation analyses have been used to identify patient subgroups to better understand observed heterogeneity in clinical outcomes and care utilization [17–19]. Among several methods for subgroup identification, latent class analysis (LCA) provides a rigorous yet intuitive statistical approach that can accommodate a large number of variables and test competing models of segmentation.

The Social Incentives to Encourage Physical Activity and Understand Predictors (STEP UP) trial was a randomized clinical trial conducted by members of our group to test the effectiveness of supportive, collaborative, and competitive social incentives within a gamification intervention to increase physical activity among overweight and obese adults from 40 U.S. states [20]. While competition was found to be most effective on average, it may not have been the best intervention for all participants. The objective of this study was to use data on demographic, behavioral and psychological characteristics collected in the STEP UP trial to identify behavioral phenotypes and evaluate differential response to the interventions.

## Methods

### Study design

This is a secondary analysis of a previously conducted randomized clinical trial (ClinicalTrials.gov identifier: NCT03311230). The clinical trial’s design, protocol, and main trial results have been previously published [20, 21]. In this study, we performed LCA of data collected during the main trial to identify participant behavioral phenotypes. We then compare changes in physical activity for each of the intervention arms relative to control within each behavioral phenotype. The University of Pennsylvania Institutional Review Board approved the study and participants provided online informed consent during the clinical trial.

Briefly, STEP UP trial was designed to test the effectiveness of supportive, competitive, and collaborative social incentives to increase physical activity in a gamification intervention that incorporated principles of behavioral economics. The supportive arm asked participants to identify a friend or family member who was encouraged to support the participant at the start of the study and received weekly reports on the participant’s performance; the competitive arm placed participants into groups of 3 and used a weekly leaderboard email to foster competition; the collaborative arm placed participants into groups of 3 and a designated member was selected each day to represent the team through their step activity.

Participants were recruited from Deloitte Consulting employees in the United States with a self-reported body mass index (BMI) of 25 or greater with a final sample of 602 employees from 40 U.S. states. During enrollment, participants were asked to complete a sociodemographic survey and a series of validated instruments to assess personality [22], risk taking behavior [23], grit [24], social support [25], exercise self-efficacy [26], mood [27], self-reported health status [28], sleep quality [29], and dietary patterns [30]. The primary outcome was change in daily steps from baseline to the 24-week intervention period. Step counts were tracked using a wrist-worn wearable device (Withings Activite Steel), which was mailed to all participants along with a replacement battery. These devices have been demonstrated to be accurate for tracking step counts [31]; devices were connected to Way to Health, a technology platform at the University of Pennsylvania used to deliver behavioral interventions [32]. The trial was conducted from February 2018 to March 2019 with a 2-week run-in period, a 24-week intervention period, and a 12-week follow-up period.

### Variable selection

Variables were selected as indicators in the LCA model based on conceptual relevance to health behavior change and known correlations to physical activity [7, 33]. We hypothesized that latent classes identified using relevant sociodemographic, psychological and behavioral variables would have differential response to the interventions. In addition, we examined the distribution of variables in our sample to exclude those without sufficient variability in distribution as they were unlikely to assist in differentiating subgroups. LCA uses discrete categorical variables as inputs [34], so all continuous variables were transformed into categorical variables based on survey scores or sample distribution as described below.

We selected variables from the following domains: 1) Demographics, which included age (grouped into 18–34 years, 35–49 years, and ≥50 years), sex, and previous use of wearable devices (yes/no). 2) Health behaviors, which included baseline physical activity and sleep quality. Baseline daily step count and coefficient of variance in baseline steps were used as measures of baseline physical activity level and consistency, respectively. They were calculated from the 2-week run-in period and converted into sample-based tertiles. Sleep quality was measured using the Pittsburg Sleep Quality Index survey and dichotomized into good sleep quality (total score <5) and poor sleep quality (total score ≥ 5) based on established interpretations of the scale [29]. 3) Personality, which included the Big Five traits of extroversion, agreeableness, conscientiousness, neuroticism, and openness [22], and grit, a measure of perseverance and passion for long-term goals [24]. 4) Behavioral perceptions, which included self-judgements of exercise self-efficacy and risk-taking specific to health and social behavior. Exercise self-efficacy was measured using the “sticking to it” factor score from the Self-Efficacy for Exercise Behaviors survey [26]. The DOSPERT survey was used to assess the likelihood to engage in risky behaviors related to health/safety and social interactions [23]. 5) Social support, which was measured using the overall score on the Medical Outcomes Study (MOS) Social Support survey and calculated as an average of subscores in the domains of emotional support, tangible support, affectionate support, and positive social interactions [25]. The overall MOS score, each of the Big Five personality traits, grit, and exercise self-efficacy were scored on a 5-point scale; variables on a 5-point scale were converted into lower (1–2.9), medium (3–3.9), and higher (4–5) levels of each trait. The DOSPERT survey uses a 7-point scale for each domain and was similarly converted into lower (1–2.9), medium (3–4.9), and higher (5–7) levels of risk-taking preference.

### Statistical analyses

We used Mplus (Version 8.2), a common software package used for LCA [35], to perform the LCA with the indicator variables detailed above. A series of models fitting two through seven classes to the baseline data were sequentially generated and compared in an iterative approach for model selection. In LCA, the best model fit is achieved by evaluating quantitative model fit indices and considering the overall interpretability of the model, which involves qualitative investigator assessments of model parsimony and class distinctiveness [36]. First, statistical model fit indices were assessed together to identify models with lower Akaike information criterion (AIC) and Bayesian information criterion (BIC) values and greater entropy values [37, 38]. To test if adding another class improved model fit, we used the parametric bootstrapped likelihood ratio test [39]. Additionally, we considered number and size of classes to maximize model interpretability and statistical power. Models with classes containing less than 5% of the total sample were eliminated.

Once the best fit model was determined, we used descriptive statistics to compare baseline variables and step count outcomes among the latent classes in R (Version 3.5.1; R Foundation for Statistical Computing). Among the indicator variables used in our LCA model, we sought to determine which variables most strongly differentiated each class from the overall study population. Thus, probability weights for each variable category were generated in Mplus and used to calculate the proportion of participants in each variable category in each class compared to the overall sample (S1 Table). We used a cutoff of 1.33 or a 33% difference from the overall sample to highlight the characteristics driving class distinction.

Similar to the STEP UP trial [20], we fit generalized mixed-effects models to compare each intervention arm to control within each latent class for the intervention and follow-up periods. Models were adjusted for baseline step count, calendar month fixed effects, and study arm. To estimate the difference in change in steps between study arms, we used a least squared means approach. We used a conservative Bonferroni adjustment for 3 comparisons using a *P* value of <0.017 for statistical significance. Regression analyses were conducted in SAS (version 9.4).

## Results

Participants had a mean (SD) age of 39 (10) years and BMI of 30 (5), 71% were male, and 64% had previously used a wearable activity tracking device. Baseline survey response rates were 100% (602/602). The mean (SD) baseline daily step count was 6204 (2646) steps and about half of the sample (49%) met criteria for poor sleep quality. Based on model fit parameters, the 3-class and 6-class model performed similarly with the lowest BIC and AIC values, respectively (S2 Table). The 3-class model was selected after considering model interpretability. The smallest class in the 3-class model still contained 20% of the sample population and clear variable patterns were qualitatively observed between classes. As the more parsimonious model, the 3-class model also offered more power to evaluate for differences between arms. All baseline variables except for the coefficient of variance in baseline steps were significantly different between the three classes (Table 1). The three classes are described in detail below. Big Five personality traits are also described relative to the sample mean consistent with trait interpretation in the psychology literature. Table 2 lists the key characteristics of each class using probability weighted variables.

**Table 1:** Baseline LCA variables in study population and by class.

	Class 1	Class 2	Class 3	P-value	Overall
	n = 328	n = 121	n = 153		n = 602
Age	40.3 (10.6)	37.8 (7.8)	36.1 (10.6)	< .001	38.72 (10.2)
Male, N (%)	230 (70.1)	99 (81.8)	98 (64.1)	0.005	427 (70.9)
Previously used a wearable, N (%)	226 (68.9)	62 (51.2)	96 (62.8)	0.002	384 (63.8)
Baseline mean daily steps	6308 (2537)	5434 (2013)	6591 (3163)	0.002	6204 (2646)
Coefficient of variance baseline steps	2.0 (0.8)	1.9 (0.8)	2.0 (0.8)	0.29	2.0 (0.8)
Extroversion (0–5, 5 = Most extroverted)	3.6 (0.8)	2.9 (0.7)	3.1 (0.8)	< .001	3.4 (0.8)
Agreeableness (0–5, 5 = Most agreeable)	4.2 (0.6)	3.8 (0.4)	3.2 (0.6)	< .001	4.0 (0.6)
Conscientiousness (0–5, 5 = Most conscientious)	4.2 (0.5)	3.8 (0.4)	3.0 (0.6)	< .001	3.9 (0.6)
Neuroticism (0–5, 5 = Most neurotic)	2.2 (0.6)	2.7 (0.6)	3.4 (0.7)	< .001	2.5 (0.8)
Openness (0–5, 5 = Most open)	3.8 (0.6)	3.3 (0.4)	3.7 (0.6)	< .001	3.7 (0.6)
ESE sticking to it (0–5, 5 = More likely to stick to exercise routine)	4.1 (0.7)	3.5 (0.6)	3.4 (0.7)	< .001	3.8 (0.7)
PSQI overall (0–15, ≥5 = Poor sleep quality)	4.4 (2.9)	4.7 (2.7)	6.4 (3.1)	< .001	5.0 (3.0)
MOS SS overall (0 to 5, 5 = More support)	4.3 (0.8)	3.9 (0.8)	3.9 (0.9)	< .001	4.1 (0.9)
DOSPERT health/safety (1 to 7, 7 = More likely to engage in risky behavior)	2.5 (1.0)	2.2 (0.8)	3.3 (1.1)	< .001	2.6 (1.1)
DOSPERT social (1 to 7, 7 = More likely to engage in risky behavior)	5.1 (1.0)	4.0 (0.8)	4.9 (0.9)	< .001	4.8 (1.0)
Grit (0–5, 5 = Extremely gritty)	4.0 (0.4)	3.6 (0.3)	3.0 (0.5)	< .001	3.7 (0.6)

Data are presented as mean (SD) of participants unless otherwise indicated.

Abbreviations: ESE, Exercise Self-Efficacy survey; PSQI, Pittsburgh Sleep Quality Index; MOS SS, Medical Outcomes Survey Social Support; DOSPERT, Domain-Specific Risk-Taking scale.

**Table 2:** Key factors driving class segmentation.

Class 1 “Extroverted and motivated”	Class 2 “Less active and less social”	Class 3 “Less motivated and at-risk”
Higher grit	Medium grit	Lower grit
Higher exercise self-efficacy	Lower exercise self-efficacy	Lower exercise self-efficacy
Higher extroversion	Lower extroversion	Lower extroversion
Higher conscientiousness	Medium conscientiousness	Lower conscientiousness
Higher openness	Lower openness	Higher neuroticism
	Lower social risk-taking	Higher health/safety risk-taking
	Lower social support	Lower social support
	Less prior wearable use	Lower agreeableness
	Lower baseline steps	Lower sleep quality
		Younger age

### Class 1- more extroverted and more motivated

Class 1 was the largest subgroup containing 54% of the study population. They were older in age (mean, 40.3 years) and 69% (226/328) had previously used a wearable device. Out of the three classes, this class had the highest scores in grit (+0.5 SD above the sample mean), conscientiousness (+0.5 SD) and exercise self-efficacy (+0.43 SD), indicating a greater degree of motivation and self-control. Other defining characteristics were higher levels of extroversion (+0.25 SD) and openness (+0.17 SD).

### Class 2- less active and less social

Class 2 comprised 20% of the study population. This class was more heavily male (82%, 99/121) with a lower baseline daily step count (mean, 5434 steps) and the largest percentage of members who had never previously used a wearable device (49%, 59/121). In contrast to class 1, members of class 2 had the lowest scores for extroversion (-0.63 SD below the mean) and openness (-0.67 SD). They had lower exercise self-efficacy but moderate levels of grit and conscientiousness. In addition, this class was characterized by lower social support and less social risk-taking behavior.

### Class 3- less motivated and at-risk

Class 3 comprised 25% of the study population. This class was youngest in age (mean, 36.1 years) and had more females (36%, 55/153) in comparison to the other classes. Members of class 3 had the highest scores for neuroticism (+1.12 SD above the mean) and lowest scores for agreeableness (-1.33 SD) and conscientiousness (-1.5 SD). Similar to class 2, members of class 3 had lower exercise self-efficacy, social support and extroversion (-0.38 SD), but tended to report more health-related risk-taking behavior. Finally, this class was notable for having the worst sleep scores among the 3 classes and were 57% more likely to have poor sleep quality than the overall sample.

### Intervention response by class

In adjusted analyses, class 1 participants had a significant change in mean daily step counts during the intervention period for only the gamification with competition arm (adjusted differences relative to control, 945 steps; 95% CI, 352–1537; *P* = .002) (Table 3). However, there was no significant change in mean daily steps relative to control for any of the gamification arms during follow-up.

**Table 3:** Adjusted model of change in mean daily step count by intervention arm for class 1 (extroverted and motivated).

Class 1 (n = 328)				
	Control n = 71	Gamification with Support n = 81	Gamification with Collaboration n = 86	Gamification with Competition n = 90
**Baseline**				
Steps per day, mean (SD)	6021 (2612)	6747 (2710)	5992 (2056)	6439 (2698)
**Main intervention period**				
Steps per day, mean (SD)	6248 (2220)	7175 (2476)	6743 (2103)	7491 (3028)
**Primary model**				
Difference relative to control and adjusted for baseline (95% CI)	-	472.1 (-72.2, 1016.4)	514 (-5.2, 1033.1)	944.8 (352.4, 1537.3)
*P* value^1^	-	0.09	0.05	0.002
**Follow-up period**				
Steps per day, mean (SD)	6187 (2437)	6584 (2566)	5850 (1442)	6718 (2792)
**Main adjusted model**				
Difference relative to control and adjusted for baseline (95% CI)	-	-38.5 (-674.1, 597.2)	-328.6 (-842.8, 185.5)	266.1 (-371.2, 903.4)
*P* value^1^	-	0.91	0.21	0.41

Abbreviations: SD, standard deviation; CI, confidence interval.

^1^Bonferroni adjusted P value <0.017 used to determine significance.

Class 2 participants in all 3 gamification arms had a significant change in mean daily steps relative to control during the intervention period that was sustained during the follow-up period (Table 4). For class 2 participants in the supportive arm, the change in mean daily steps relative to control was 1172 (95% CI, 363–1980; *P* = 0.005) during the intervention period and 1061 (95% CI, 343–1669; *P* = 0.003) during the follow-up period. For class 2 participants in the collaborative arm, the change in mean daily steps relative to control was 1119 (95% CI, 319–1919; *P* = 0.006) during the intervention period and 1044 (95% CI 413–1675; *P* = 0.001) during the follow-up period. For class 2 participants in the competitive arm, the change in mean daily steps relative to control was 1179 (95% CI, 400–1957; *P* = 0.003) during the intervention period and 941 (95% CI, 269–1614; *P* = 0.006) during the follow-up period.

**Table 4:** Adjusted model of change in mean daily step count by intervention arm for class 2 (less active and less social).

Class 2 (n = 121)				
	Control n = 33	Gamification with Support n = 30	Gamification with Collaboration n = 29	Gamification with Competition n = 29
**Baseline**				
Steps per day, mean (SD)	5588 (1844)	5407 (1995)	5098 (1964)	5624 (2306)
**Main intervention period**				
Steps per day, mean (SD)	5488 (1425)	6556 (2393)	6333 (2290)	6693 (2404)
**Primary model**				
Difference relative to control and adjusted for baseline (95% CI)	-	1171.7 (363.3, 1980.1)	1119.4 (319.8, 1919.1)	1178.5 (400.3, 1956.6)
*P* value^1^	-	0.005	0.006	0.003
**Follow-up period**				
Steps per day, mean (SD)	5039 (1062)	5974 (1998)	5888 (1738)	6024 (1973)
**Main adjusted model**				
Difference relative to control and adjusted for baseline (95% CI)	-	1006.1 (343, 1669.2)	1043.7 (412.9, 1674.6)	941.4 (268.7, 1614)
*P* value^1^	-	0.003	0.001	0.006

Abbreviations: SD, standard deviation; CI, confidence interval.

^1^Bonferroni adjusted P value <0.017 used to determine significance.

Among participants in class 3, there was no significant change in mean daily steps relative to control for any of the gamification arms during the intervention or follow-up periods. (Table 5).

**Table 5:** Adjusted model of change in mean daily step count by intervention arm for class 3 (less motivated and at-risk).

Class 3 (n = 153)				
	Control n = 47	Gamification with Support n = 40	Gamification with Collaboration n = 35	Gamification with Competition n = 31
**Baseline**				
Steps per day, mean (SD)	6533 (3073)	6053 (2513)	7282 (3628)	6592 (3482)
**Main intervention period**				
Steps per day, mean (SD)	6505 (2366)	6884 (2267)	7387 (2286)	7009 (3102)
**Primary model**				
Difference relative to control and adjusted for baseline (95% CI)	-	620.3 (-160, 1400.7)	545.4 (-247.4, 1338.2)	466.2 (-348.1, 1280.4)
*P* value^1^	-	0.12	0.18	0.26
**Follow-up period**				
Steps per day, mean (SD)	6068 (2063)	6467 (2006)	6624 (1908)	6758 (2945)
**Main adjusted model**				
Difference relative to control and adjusted for baseline (95% CI)	-	593.2 (-152.4, 1338.8)	273.9 (-472.2, 1019.9)	662.6 (- 205.4, 1530.6)
*P* value^1^	-	0.12	0.47	0.13

Abbreviations: SD, standard deviation; CI, confidence interval.

^1^Bonferroni adjusted P value <0.017 used to determine significance.

## Discussion

In this secondary analysis of a randomized clinical trial, we demonstrated that baseline demographic, behavioral and psychological characteristics identified groups of participants with behavioral phenotypes that had differential response to the physical activity interventions. Rather than examining predictors in isolation, we used latent class analysis to reveal groups defined by co-occurring traits and behavioral patterns. These variable groupings constitute behavioral phenotypes that could provide a framework for selecting target populations of behavioral interventions [40]. While the main trial analyses showed that competition was most effective overall, our segmentation reveals substantial variations in intervention response by behavioral phenotype.

Class 1 participants performed best in the competitive arm and accounted for over half of the study population. However, they did not maintain their physical activity during follow-up despite high levels of exercise self-efficacy, conscientiousness and grit. It may be that these traits enable individuals to perform favorably under social influence and comparison but have a smaller influence on habit formation. Sustaining behavior change is known to be challenging after incentives are withdrawn [41]. Ongoing incentive programs may be necessary to support many individuals, including those with high baseline motivation and self-control, and should be explored in future work.

Notably, class 2 participants significantly increased and sustained their physical activity in all three gamification arms through the follow-up period. Class 2 may represent the ideal participant phenotype for a remotely monitored gamification intervention that uses behavioral economics and social influence to promote physical activity. Since members of this class were less extroverted, less open, less likely to engage in social risk taking, and reported less social support, offering structured social incentives may be particularly impactful. Additionally, this group was less active and reported lower exercise self-efficacy at baseline, but had moderate levels of conscientiousness and grit, traits theorized to be protective for health [14]. The persistence of intervention effects in the follow-up period and similar outcomes seen across the intervention arms indicates that interventions may have facilitated habit formation for regular physical activity among individuals within this phenotype.

On the other hand, class 3 participants did not appear to benefit from any of the interventions. This group demonstrated traits seen in Type D personality or distressed personality type, a well-established phenotype characterized by negative affect and social inhibition which correlates with low extraversion, low conscientiousness and high neuroticism [42]. This personality type has been associated with a range of negative mental and physical health outcomes, felt to be mediated by poor health behaviors and maladaptive coping mechanisms [43]. Thus, the findings of worse sleep quality and more health-related risk-taking behaviors in this group is also consistent. Individuals with these characteristics could require more intensive cognitively designed interventions to support behavior change through mechanisms such as improving self-efficacy [44].

This is one of the first studies to use LCA to identify behavioral phenotypes and evaluate variations in participant response in a randomized clinical trial for increasing physical activity. Prior studies in this area have used recursive partitioning methods which uses cutpoints to split predictor variables [15, 45]. However, without careful application and parameter tuning, these flexible methods can be prone to overfitting, resulting in unstable decision trees and classification structures [46]. LCA represents an alternative approach to segmentation that can be evaluated by likelihood-based model fit indices with growing applications to healthcare in the era of personalized medicine. In the context of health behavior interventions, it is well-suited to synthesize the large number and wide range of potential predictors of success and the complex interactions between them that remain poorly understood and likely vary between populations. For example, LCA has been used to identify subgroups among African American and Chinese American patients with low rates of colon cancer screening based on sociodemographics and an expanded set of psychosocial risk factors including perceived barriers, health beliefs and cultural values [47, 48]. However, prospective studies that test targeted and behaviorally designed interventions are lacking.

Among digital health interventions for physical activity, there is evidence that leveraging social networks and incorporating features such as social support and social comparison in addition to self-monitoring can enhance engagement and retention [49]. At the same time, participants have expressed a range of preferences and concerns regarding these intervention features. In our study, class 2 participants increased their physical activity in all 3 intervention arms, suggesting that the type of social incentive used was not a major determinant of intervention response for this subgroup. Further studies are needed to explore differential response to social incentives in behavioral interventions, the role of stated participant preferences, and whether an ideal participant phenotype exists for specific incentives.

### Limitations

This study has several limitations. First, it is a secondary analysis of a randomized clinical trial. Our main objective was to use LCA to identify subgroups in the study population as a framework for understanding differential intervention effects [50]. The main trial was not designed to detect differences in step count between intervention and control among three latent classes so the analyses in the current study may be underpowered. Second, LCA only reveals patterns in variation present at baseline without consideration of the outcome. This approach does not formally test how to target identified subgroups or compare the relative benefits of targeting techniques, which will require alternative statistical prediction models and prospective studies. Third, this population of overweight and obese adults was from a large U.S. consulting firm, limiting the generalizability of our findings. Nonetheless, the distinct phenotypes present in this population suggest the importance of accounting for individual differences in personality, behavioral tendencies and social resources when designing and evaluating wellness programs.

## Conclusions

We identified three behavioral phenotypes among overweight and obese adult participants of a randomized clinical trial of a gamification intervention to increase physical activity. The phenotypes were characterized by a combination of data on demographics, health behaviors and psychological measures and were associated with different intervention outcomes. These findings demonstrated that a behavioral phenotyping approach can reveal differences in intervention response and identify those who are most likely to benefit. Future studies should establish and validate classifications of behavioral phenotypes in broader populations and formally test interventions targeted to specific participant phenotypes.

## Supporting information

S1 Table Variable weights by class. (DOCX)

S2 Table Fit statistics and number of individuals per class for latent class models for two to seven classes. (DOCX)

S1 Dataset Raw data used for STEP UP LCA analyses. (XLSX)

## References

[1] McGinnis JM, Williams-Russo P, Knickman JR. The Case For More Active Policy Attention To Health Promotion. Health Affairs. 2002;21(2):78–93. 10.1377/hlthaff.21.2.78 11900188

[2] Schroeder SA. We Can Do Better—Improving the Health of the American People. New England Journal of Medicine. 2007;357(12):1221–8. 10.1056/NEJMsa073350 . 17881753

[3] Teasdale N, Elhussein A, Butcher F, Piernas C, Cowburn G, Hartmann-Boyce J, et al Systematic review and meta-analysis of remotely delivered interventions using self-monitoring or tailored feedback to change dietary behavior. Am J Clin Nutr. 2018;107(2):247–56. 10.1093/ajcn/nqx048 . 29529158 PMC5875102

[4] Fanshawe TR, Hartmann‐Boyce J, Perera R, Lindson N. Competitions for smoking cessation. Cochrane Database of Systematic Reviews. 2019;(2). 10.1002/14651858.CD013272 . PMC6953205 30784046

[5] Barte JCM, Wendel-Vos GCW. A Systematic Review of Financial Incentives for Physical Activity: The Effects on Physical Activity and Related Outcomes. Behavioral Medicine. 2017;43(2):79–90. 10.1080/08964289.2015.1074880 26431076

[6] Bauman AE, Sallis JF, Dzewaltowski DA, Owen N. Toward a better understanding of the influences on physical activity: The role of determinants, correlates, causal variables, mediators, moderators, and confounders. American Journal of Preventive Medicine. 2002;23(2):5–14. 10.1016/S0749-3797(02)00469-5 12133733

[7] Rhodes RE, Janssen I, Bredin SSD, Warburton DER, Bauman A. Physical activity: Health impact, prevalence, correlates and interventions. Psychology & Health. 2017;32(8):942–75. 10.1080/08870446.2017.1325486 28554222

[8] Conn VS, Hafdahl AR, Mehr DR. Interventions to increase physical activity among healthy adults: meta-analysis of outcomes. Am J Public Health. 2011;101(4):751–8. Epub 2011/02/17. 10.2105/AJPH.2010.194381 . 21330590 PMC3052337

[9] Murray JM, Brennan SF, French DP, Patterson CC, Kee F, Hunter RF. Effectiveness of physical activity interventions in achieving behaviour change maintenance in young and middle aged adults: A systematic review and meta-analysis. Social Science & Medicine. 2017;192:125–33. 10.1016/j.socscimed.2017.09.021 28965003

[10] Simmons RK, van Sluijs EMF, Hardeman W, Sutton S, Griffin SJ, the ProActive project t. Who will increase their physical activity? Predictors of change in objectively measured physical activity over 12 months in the ProActive cohort. BMC Public Health. 2010;10(1):226 10.1186/1471-2458-10-226 20433700 PMC2873588

[11] Westland H, Sluiter J, te Dorsthorst S, Schröder CD, Trappenburg JCA, Vervoort SCJM, et al Patients’ experiences with a behaviour change intervention to enhance physical activity in primary care: A mixed methods study. PLOS ONE. 2019;14(2):e0212169 10.1371/journal.pone.0212169 30753213 PMC6372184

[12] Edlind M, Mitra N, Grande D, Barg FK, Carter T, Turr L, et al Why Effective Interventions Do Not Work for All Patients: Exploring Variation in Response to a Chronic Disease Management Intervention. Med Care. 2018;56(8):719–26. Epub 2018/06/26. 10.1097/MLR.0000000000000939 29939912 PMC6041152

[13] Scheier MF, Helgeson VS, Schulz R, Colvin S, Berga SL, Knapp J, et al Moderators of Interventions Designed to Enhance Physical and Psychological Functioning Among Younger Women With Early-Stage Breast Cancer. Journal of Clinical Oncology. 2007;25(36):5710–4. 10.1200/JCO.2007.11.7093 . 17998547

[14] Chapman BP, Roberts B, Duberstein P. Personality and longevity: knowns, unknowns, and implications for public health and personalized medicine. J Aging Res. 2011;2011:759170 Epub 2011/07/19. 10.4061/2011/759170 21766032 PMC3134197

[15] Boslaugh SE, Kreuter MW, Nicholson RA, Naleid K. Comparing demographic, health status and psychosocial strategies of audience segmentation to promote physical activity. Health Education Research. 2004;20(4):430–8. 10.1093/her/cyg138 15572439

[16] Forthofer MS, Bryant CA. Using Audience-Segmentation Techniques to Tailor Health Behavior Change Strategies. American Journal of Health Behavior. 2000;24(1):36–43. 10.5993/AJHB.24.1.6

[17] Davis AC, Shen E, Shah NR, Glenn BA, Ponce N, Telesca D, et al Segmentation of High-Cost Adults in an Integrated Healthcare System Based on Empirical Clustering of Acute and Chronic Conditions. Journal of General Internal Medicine. 2018;33(12):2171–9. 10.1007/s11606-018-4626-0 30182326 PMC6258619

[18] Calfee CS, Delucchi K, Parsons PE, Thompson BT, Ware LB, Matthay MA, et al Subphenotypes in acute respiratory distress syndrome: latent class analysis of data from two randomised controlled trials. Lancet Respir Med. 2014;2(8):611–20. Epub 2014/05/19. 10.1016/S2213-2600(14)70097-9 . 24853585 PMC4154544

[19] Keeney T, Belanger E, Jones RN, Joyce NR, Meyers DJ, Mor V. High-Need Phenotypes in Medicare Beneficiaries: Drivers of Variation in Utilization and Outcomes. Journal of the American Geriatrics Society. 2019;0(0). Epub 2019 Aug 27. 10.1111/jgs.16146 31454082 PMC6952536

[20] Patel MS, Small DS, Harrison JD, Fortunato MP, Oon AL, Rareshide CAL, et al Effectiveness of Behaviorally Designed Gamification Interventions With Social Incentives for Increasing Physical Activity Among Overweight and Obese Adults Across the United States: The STEP UP Randomized Clinical Trial. JAMA Internal Medicine. 2019:1–9. 10.1001/jamainternmed.2019.3505 31498375 PMC6735420

[21] Harrison JD, Jones JM, Small DS, Rareshide CAL, Szwartz G, Steier D, et al Social incentives to encourage physical activity and understand predictors (STEP UP): Design and rationale of a randomized trial among overweight and obese adults across the United States. Contemporary Clinical Trials. 2019;80:55–60. 10.1016/j.cct.2019.04.001 30954675

[22] John OP, Srivastava S. The Big Five Trait taxonomy: History, measurement, and theoretical perspectives Handbook of personality: Theory and research, 2nd ed. New York, NY, US: Guilford Press; 1999 p. 102–38.

[23] Blais A-R, Weber EU. A Domain-Specific Risk-Taking (DOSPERT) scale for adult populations. Judgment and Decision Making. 2006;1(1):33–47.

[24] Duckworth AL, Peterson C, Matthews MD, Kelly DR. Grit: Perseverance and passion for long-term goals. Journal of Personality and Social Psychology. 2007;92(6):1087–101. 10.1037/0022-3514.92.6.1087 17547490

[25] Sherbourne CD, Stewart AL. The MOS social support survey. Social Science & Medicine. 1991;32(6):705–14. 10.1016/0277-9536(91)90150-B 2035047

[26] Sallis JF, Pinski RB, Grossman RM, Patterson TL, Nader PR. The development of self-efficacy scales for healthrelated diet and exercise behaviors. Health Education Research. 1988;3(3):283–92. 10.1093/her/3.3.283

[27] Kroenke K, Spitzer RL, Williams JB. The PHQ-9: validity of a brief depression severity measure. Journal of general internal medicine. 2001;16(9):606–13. 10.1046/j.1525-1497.2001.016009606.x . 11556941 PMC1495268

[28] Herdman M, Gudex C, Lloyd A, Janssen M, Kind P, Parkin D, et al Development and preliminary testing of the new five-level version of EQ-5D (EQ-5D-5L). Quality of Life Research. 2011;20(10):1727–36. 10.1007/s11136-011-9903-x 21479777 PMC3220807

[29] Buysse DJ, Reynolds CF, Monk TH, Berman SR, Kupfer DJ. The Pittsburgh sleep quality index: A new instrument for psychiatric practice and research. Psychiatry Research. 1989;28(2):193–213. 10.1016/0165-1781(89)90047-4 2748771

[30] Lean MEJ, Anderson AS, Morrison C, Currall J. Evaluation of a Dietary Targets Monitor. European Journal of Clinical Nutrition. 2003;57(5):667–73. 10.1038/sj.ejcn.1601596 12771967

[31] Case MA, Burwick HA, Volpp KG, Patel MS. Accuracy of Smartphone Applications and Wearable Devices for Tracking Physical Activity Data. JAMA. 2015;313(6):625–6. 10.1001/jama.2014.17841 25668268

[32] Asch DA, Volpp KG. On the Way to Health. LDI Issue Brief. 2012;17(9):1–4. Epub 2012/09/01. . 22934330

[33] Rhodes RE, Smith NEI. Personality correlates of physical activity: a review and meta-analysis. British Journal of Sports Medicine. 2006;40(12):958–65. 10.1136/bjsm.2006.028860 17124108 PMC2577457

[34] Oberski D. Mixture Models: Latent Profile and Latent Class Analysis In: Robertson J, Kaptein M, editors. Modern Statistical Methods for HCI. Cham: Springer International Publishing; 2016 p. 275–87.

[35] Mplus User's Guide. Los Angeles, CA: Muthén & Muthén; 2007.

[36] Collins LM, Lanza ST. Latent Class and Latent Transition Analysis: With Applications in the Social, Behavioral, and Health Sciences: John Wiley and Sons Inc.; 2010. 295 p.

[37] Nylund KL, Asparouhov T, Muthén BO. Deciding on the Number of Classes in Latent Class Analysis and Growth Mixture Modeling: A Monte Carlo Simulation Study. Structural Equation Modeling: A Multidisciplinary Journal. 2007;14(4):535–69. 10.1080/10705510701575396

[38] Vrieze SI. Model selection and psychological theory: a discussion of the differences between the Akaike information criterion (AIC) and the Bayesian information criterion (BIC). Psychol Methods. 2012;17(2):228–43. Epub 2012/02/09. 10.1037/a0027127 22309957 PMC3366160

[39] Lo Y, Mendell NR, Rubin DB. Testing the number of components in a normal mixture. Biometrika. 2001;88(3):767–78. 10.1093/biomet/88.3.767

[40] Hardcastle SJ, Hagger MS. Psychographic Profiling for Effective Health Behavior Change Interventions. Frontiers in Psychology. 2016;6(1988). 10.3389/fpsyg.2015.01988 26779094 PMC4701903

[41] Strohacker K, Galarraga O, Williams DM. The Impact of Incentives on Exercise Behavior: A Systematic Review of Randomized Controlled Trials. Annals of Behavioral Medicine. 2013;48(1):92–9. 10.1007/s12160-013-9577-4 24307474 PMC4412849

[42] Chapman BP, Duberstein PR, Lyness JM. The Distressed personality type: replicability and general health associations. European Journal of Personality. 2007;21(7):911–29. 10.1002/per.645

[43] Mols F, Denollet J. Type D personality in the general population: a systematic review of health status, mechanisms of disease, and work-related problems. Health and Quality of Life Outcomes. 2010;8(1):9 10.1186/1477-7525-8-9 20096129 PMC2822747

[44] Franks P, Chapman B, Duberstein P, Jerant A. Five factor model personality factors moderated the effects of an intervention to enhance chronic disease management self-efficacy. Br J Health Psychol. 2009;14(Pt 3):473–87. Epub 2008/09/24. 10.1348/135910708X360700 18808733 PMC2745728

[45] King AC, Marcus B, Ahn D, Dunn AL, Rejeski WJ, Sallis JF, et al Identifying subgroups that succeed or fail with three levels of physical activity intervention: The activity counseling trial. Health Psychology. 2006;25(3):336–47. 10.1037/0278-6133.25.3.336 16719605

[46] Strobl C, Malley J, Tutz G. An introduction to recursive partitioning: rationale, application, and characteristics of classification and regression trees, bagging, and random forests. Psychol Methods. 2009;14(4):323–48. 10.1037/a0016973 . 19968396 PMC2927982

[47] BeLue R, Menon U, Kinney AY, Szalacha LA. Psychosocial risk profiles among black male veterans administration patients non-adherent with colorectal cancer screening. Psycho-Oncology. 2011;20(11):1151–60. 10.1002/pon.1838 20928929 PMC3096702

[48] Strong C, Ji CS, Liang W, Ma G, Brown R, Wang JH-y. Heterogeneous demographic and cultural profiles of Chinese American patients nonadherent to colorectal cancer screening: a latent class analysis. Cancer Nurs. 2014;37(2):106–13. 10.1097/NCC.0b013e3182888b5b . 23519040 PMC3692589

[49] Tong HL, Laranjo L. The use of social features in mobile health interventions to promote physical activity: a systematic review. npj Digital Medicine. 2018;1(1):43 10.1038/s41746-018-0051-3 31304323 PMC6550193

[50] Lanza ST, Rhoades BL. Latent class analysis: an alternative perspective on subgroup analysis in prevention and treatment. Prev Sci. 2013;14(2):157–68. 10.1007/s11121-011-0201-1 . 21318625 PMC3173585

