# Supplementary material for: Association between behavioral phenotypes and response to a physical activity intervention using gamification and social incentives: Secondary analysis of the STEP UP randomized clinical trial
Source: PLoS One. 2020 Oct 14;15(10):e0239288. doi: 10.1371/journal.pone.0239288 (PMC7556484; doi:10.1371/journal.pone.0239288)
Supplement: S1 Table — (DOCX) [file pone.0239288.s001.docx]

| **S1 Table. Variable weights by class** | |  |  |  |  |  |  |
| --- | --- | --- | --- | --- | --- | --- | --- |
|  |  |  |  |  |  |  |  |
| **Baseline variables** | **Proportion of Overall Sample** | **Class 1** | | **Class 2** | | **Class 3** | |
|  |  | **Proportion of Class Sample** | **Class Proportion Relative to Overall Proportion** | **Proportion of Class Sample** | **Class Proportion Relative to Overall Proportion** | **Proportion of Class Sample** | **Class Proportion Relative to Overall Proportion** |
| Baseline Steps |  |  |  |  |  |  |  |
| Low (bottom third) | 0.33 | 0.31 | 0.92 | 0.44 | 1.33 | 0.30 | 0.92 |
| Med | 0.33 | 0.34 | 1.01 | 0.34 | 1.02 | 0.32 | 0.96 |
| High (top third) | 0.33 | 0.36 | 1.07 | 0.22 | 0.66 | 0.37 | 1.12 |
| Coefficient of variance baseline steps |  |  |  |  |  |  |  |
| Low (bottom third) | 0.33 | 0.34 | 1.02 | 0.36 | 1.08 | 0.30 | 0.90 |
| Med | 0.33 | 0.36 | 1.06 | 0.28 | 0.82 | 0.33 | 1.00 |
| High (top third) | 0.33 | 0.31 | 0.92 | 0.37 | 1.09 | 0.37 | 1.10 |
| Age |  |  |  |  |  |  |  |
| Low, 18-34 | 0.41 | 0.34 | 0.83 | 0.40 | 0.97 | 0.56 | 1.38 |
| Med, 35-50 | 0.43 | 0.46 | 1.08 | 0.51 | 1.21 | 0.28 | 0.67 |
| High, >50 | 0.17 | 0.20 | 1.20 | 0.09 | 0.55 | 0.16 | 0.92 |
| Gender |  |  |  |  |  |  |  |
| Female | 0.29 | 0.29 | 1.00 | 0.22 | 0.75 | 0.34 | 1.18 |
| Male | 0.71 | 0.71 | 1.00 | 0.78 | 1.10 | 0.66 | 0.93 |
| ESE sticking to it |  |  |  |  |  |  |  |
| Low, 1-2.9 | 0.13 | 0.05 | 0.40 | 0.21 | 1.59 | 0.24 | 1.80 |
| Med, 3-3.9 | 0.43 | 0.34 | 0.79 | 0.56 | 1.30 | 0.53 | 1.22 |
| High, 4-5 | 0.44 | 0.61 | 1.40 | 0.23 | 0.53 | 0.24 | 0.54 |
| PSQI quality |  |  |  |  |  |  |  |
| Bad, 1-4 | 0.37 | 0.28 | 0.76 | 0.33 | 0.91 | 0.57 | 1.57 |
| Good, ≥5 | 0.64 | 0.72 | 1.14 | 0.67 | 1.05 | 0.43 | 0.67 |
| Previously used a wearable |  |  |  |  |  |  |  |
| No | 0.36 | 0.32 | 0.88 | 0.48 | 1.33 | 0.36 | 0.99 |
| Yes | 0.64 | 0.68 | 1.07 | 0.52 | 0.81 | 0.64 | 1.01 |
| Extroversion |  |  |  |  |  |  |  |
| Low, 1-2.9 | 0.32 | 0.20 | 0.63 | 0.43 | 1.36 | 0.48 | 1.50 |
| Med, 3-3.9 | 0.43 | 0.44 | 1.04 | 0.52 | 1.23 | 0.31 | 0.74 |
| High, 4-5 | 0.26 | 0.36 | 1.39 | 0.05 | 0.19 | 0.21 | 0.82 |
| Agreeableness |  |  |  |  |  |  |  |
| Low, 1-2.9 | 0.05 | 0.03 | 0.58 | 0.03 | 0.57 | 0.12 | 2.23 |
| Med, 3-3.9 | 0.42 | 0.28 | 0.67 | 0.55 | 1.31 | 0.60 | 1.44 |
| High, 4-5 | 0.53 | 0.69 | 1.30 | 0.42 | 0.80 | 0.28 | 0.53 |
| Conscientiousness |  |  |  |  |  |  |  |
| Low, 1-2.9 | 0.06 | 0.00 | 0.05 | 0.01 | 0.19 | 0.23 | 3.62 |
| Med, 3-3.9 | 0.44 | 0.25 | 0.58 | 0.70 | 1.58 | 0.63 | 1.44 |
| High, 4-5 | 0.50 | 0.74 | 1.49 | 0.29 | 0.59 | 0.14 | 0.29 |
| Neuroticism |  |  |  |  |  |  |  |
| Low, 1-2.9 | 0.71 | 0.90 | 1.28 | 0.63 | 0.89 | 0.35 | 0.50 |
| High, 4-5 | 0.29 | 0.10 | 0.33 | 0.37 | 1.26 | 0.65 | 2.21 |
| Openness |  |  |  |  |  |  |  |
| Low, 1-2.9 | 0.11 | 0.07 | 0.62 | 0.22 | 2.03 | 0.11 | 1.00 |
| Med, 3-3.9 | 0.58 | 0.49 | 0.86 | 0.79 | 1.37 | 0.59 | 1.02 |
| High, 4-5 | 0.32 | 0.44 | 1.38 | 0.00 | 0.00 | 0.31 | 0.97 |
| MOS SS overall |  |  |  |  |  |  |  |
| Low, 1-2.9 | 0.13 | 0.07 | 0.59 | 0.18 | 1.46 | 0.19 | 1.48 |
| Med, 3-3.9 | 0.26 | 0.20 | 0.79 | 0.37 | 1.44 | 0.29 | 1.11 |
| High, 4-5 | 0.62 | 0.72 | 1.17 | 0.45 | 0.72 | 0.53 | 0.86 |
| DOSPERT health safety |  |  |  |  |  |  |  |
| Low, 1-2.9 | 0.63 | 0.69 | 1.09 | 0.77 | 1.22 | 0.40 | 0.63 |
| Med, 3-4.9 | 0.34 | 0.30 | 0.87 | 0.23 | 0.66 | 0.52 | 1.52 |
| High, 5-7 | 0.03 | 0.01 | 0.37 | 0.00 | 0.00 | 0.08 | 3.04 |
| DOSPERT social |  |  |  |  |  |  |  |
| Low, 1-2.9 | 0.04 | 0.03 | 0.68 | 0.10 | 2.40 | 0.02 | 0.58 |
| Med, 3-4.9 | 0.47 | 0.35 | 0.74 | 0.79 | 1.67 | 0.48 | 1.01 |
| High, 5-7 | 0.49 | 0.62 | 1.27 | 0.12 | 0.24 | 0.50 | 1.02 |
| Grit |  |  |  |  |  |  |  |
| Low, 1-2.9 | 0.12 | 0.00 | 0.03 | 0.01 | 0.04 | 0.46 | 3.78 |
| Med, 3-3.9 | 0.51 | 0.36 | 0.71 | 0.86 | 1.70 | 0.54 | 1.08 |
| High, 4-5 | 0.37 | 0.64 | 1.71 | 0.14 | 0.37 | 0.00 | 0.00 |

Abbreviations: ESE, Exercise Self-Efficacy survey; PSQI, Pittsburgh Sleep Quality Index; MOS SS, Medical Outcomes Survey Social Support; DOSPERT, Domain-Specific Risk-Taking scale.
