# Supplementary material for: Association between behavioral phenotypes and response to a physical activity intervention using gamification and social incentives: Secondary analysis of the STEP UP randomized clinical trial
Source: PLoS One. 2020 Oct 14;15(10):e0239288. doi: 10.1371/journal.pone.0239288 (PMC7556484; doi:10.1371/journal.pone.0239288)
Supplement: S2 Table — (DOCX) [file pone.0239288.s002.docx]

**S2 Table. Fit statistics and number of individuals per class for latent class models for two to seven classes.**

| **Number of classes** | **AIC** | **BIC^a^** | **Entropy^b^** | **N_1_** | **N_2_** | **N_3_** | **N_4_** | **N_5_** | **N_6_** | **N_7_** | **p-value^c^** |
| --- | --- | --- | --- | --- | --- | --- | --- | --- | --- | --- | --- |
| 2 | 16396.1 | 16466 | 0.709 | 335 | 267 |  |  |  |  |  | <0.001 |
| 3 | 16331.6 | 16437 | 0.751 | 328 | 121 | 153 |  |  |  |  | <0.001 |
| 4 | 16303.2 | 16444.1 | 0.751 | 112 | 299 | 73 | 118 |  |  |  | <0.001 |
| 5 | 16288.3 | 16464.8 | 0.731 | 107 | 54 | 132 | 203 | 106 |  |  | 0.03 |
| 6 | 16276.5 | 16488.4 | 0.783 | 223 | 134 | 109 | 43 | 43 | 50 |  | 0.0128 |
| 7 | 16284.5 | 16532.1 | 0.764 | 77 | 103 | 134 | 38 | 88 | 26 | 136 | 1 |

Abbreviations: AIC, Akaike information criterion; BIC, Bayesian information criterion.

^a^ Adjusted for sample size

^b^ Entropy is a measure of class separation with higher values indicating better separation.

^c^ By the parametric bootstrapped likelihood ratio test, testing whether increasing the number of classes provides improved model fit compared to a model using one fewer class.
