# Supplementary material for: Association between behavioral phenotypes and response to a physical activity intervention using gamification and social incentives: Secondary analysis of the STEP UP randomized clinical trial
Source: PLoS One. 2020 Oct 14;15(10):e0239288. doi: 10.1371/journal.pone.0239288 (PMC7556484; doi:10.1371/journal.pone.0239288)
Supplement: Attachment — Submitted filename: Response to Reviewers.docx [file pone.0239288.s004.docx]

**Response to Reviewers**

**Re: Association between Behavioral Phenotypes and Response to a Physical Activity Intervention Using Gamification and Social Incentives: Secondary Analysis of the STEP UP Randomized Clinical Trial**

Reviewer #1: This manuscript presents a secondary analysis of data from a randomized clinical trial using latent class analysis to determine whether subgroups of participants responded different to the physical activity intervention. Overall, the methods and analytical approach are sound, and the results are interesting. The manuscript is well-written and succinct. I have a few recommendations for changes that I believe would further enhance the manuscript.

General comments:

1. I can appreciate that coming up with succinct, accurate names for each of the groups is a challenge. I thought the name for Class 2 was a good reflection of its defining characteristics. However, for Class 1 something that highlights their grit and conscientiousness might be more appropriate, as motivation was not directly assessed and extroversion was not as far above the mean as some of the other variables. For Class 3, again motivation was not assessed and the term “at-risk” is quite vague – at-risk for low physical activity? For poor mental health? I don’t have a suggestion for a perfect name for these groups, but I would encourage the authors to consider whether the current names can be improved.

We named the three latent classes to convey a general sense of the defining characteristics of each group based on the variable weights (S1 Table) rather than comparisons of mean variable scores alone. For example, participants in Class 1 were 39% more likely to have a high extroversion score compared to overall sample. While motivation was not directly assessed, we use the descriptor of “motivated” for Class 1 and “less motivated” for Class 3 to highlight the clustering of exercise self-efficacy, conscientiousness and grit which were more likely to be high in Class 1 and low in Class 3. In naming the classes, we felt that motivation could succinctly capture these patterns. Similarly, the decision to name Class 3 “at-risk” is intended to capture a combination of traits that may put this group at higher risk for poor health across several domains—specifically the higher proportion of participants in Class 3 with high neuroticism scores, low social support scores, low sleep quality scores, and high health and safety related risk-taking behavior scores.

2. I also appreciated that the authors did not overstate their findings and kept the discussion limited to this specific trial (e.g. p. 15, lines 294-6, “the ideal participant phenotype for a remotely monitored gamification intervention that uses behavioral economics and social influence to promote physical activity.”). On the other hand, this is a very specific intervention type, and readers may wonder whether these findings would be relevant to other types of behavioral interventions. Although there may be a limited number of studies that have used this exact approach (e.g., LCA in the context of physical activity RCTs), there are a variety of studies that have examined how subgroups of the total participant population respond differently to interventions. I would like to see the discussion incorporate some additional literature to put these results in the broader context of what we already know.

There is very limited literature on prospective control trials of health behavior interventions targeted to subgroups. As we emphasized in the introduction section, studies have mostly looked at differential response via traditional post-hoc subgroup analyses. In general, responders cannot be easily or reliably differentiated from non-responders based on common sociodemographic variables, clinical predictors such as health status, or even measures of motivation such as patient activation. Despite the specificity of this physical activity intervention, we do believe that our findings are relevant to other behavioral interventions since many of the barriers and challenges to health behavior change are similar and related to individual level characteristics. We have expanded our discussion section and include examples of studies on colon cancer screening behavior to address this as detailed below.

3. Similar to the comment above, I think it would be helpful to incorporate some additional discussion about how these types of studies can be used to enhance future interventions. The authors provide some general comments about targeting subgroups, but from a practical perspective, how might this be done? Based on this study and others like it, are there specific variables that should be consistently measured at baseline and used to determine what types of support participants may need? Are there evidence-based examples of different types of support being effective for subgroups of participants?

As we acknowledge in the limitations, this study was a secondary analysis of a specific physical activity trial and we were not able to evaluate specific methods for targeting subgroups. We highlight LCA as a potential strategy to identify relevant subgroups using a combination of sociodemographic, behavioral and psychological variables. More research is needed to determine which variables should be measured at baseline and whether these are specific to the health behavior and intervention of interest. Our findings suggest that adding psychological variables to traditional sociodemographic data provides value to identifying subgroups with differential response to a physical activity intervention. Recent studies on colon cancer screening behavior also suggest that measures of psychosocial risk can meaningfully distinguish subgroups among relatively demographically homogenous populations.

We have added text on page 16, lines 329-333 as follows: For example, LCA has been used to identify subgroups among African American and Chinese American patients with low rates of colon cancer screening based on sociodemographics and an expanded set of psychosocial risk factors including perceived barriers, health beliefs and cultural values [47, 48]. However, prospective studies that test targeted and behaviorally designed interventions are lacking.

In addition, we believe that our findings have specific relevance to the growing number of physical activity interventions that incorporate social incentives. Though these incentives can be effective, participants have expressed mixed preferences. Interestingly, our findings indicate that the type of social incentive used may be relevant for some participant subgroups but not others. Thus, targeting social incentives may be more complex than matching to stated user preferences and more research is needed to understand the underlying participant differences that may influence intervention response.

We have added text on page 17, lines 334-342 as follows: Among digital health interventions for physical activity, there is evidence that leveraging social networks and incorporating features such as social support and social comparison in addition to self-monitoring can enhance engagement and retention [49]. At the same time, participants have expressed a range of preferences and concerns regarding these intervention features. In our study, class 2 participants increased their physical activity in all 3 intervention arms, suggesting that the type of social incentive used was not a major determinant of intervention response for this subgroup. Further studies are needed to explore differential response to social incentives in behavioral interventions, the role of stated participant preferences, and whether an ideal participant phenotype exists for specific incentives.

Specific comments:

p. 5, line 107: was the follow-up measure taken at the end of the 24-week intervention period? Were step counts averaged across a week at each time point?

The follow-up period lasted for 12 weeks after the end of the 24-week intervention period. Step counts were treated as a continuous variable for each patient on each day of the study (including the intervention and follow-up period).

p. 5, line 107-8: Did all participants wear the same wearable device? What device was it?

Yes, all participants used the same wrist-worn wearable device. We have modified the text on page 5, lines 108-110 as follows: Step counts were tracked using a wrist-worn wearable device (Withings Activite Steel), which was mailed to all participants along with a replacement battery.

The manuscript sometimes uses “randomized clinical trial” and other times “randomized control trial” – be consistent throughout.

We have changed the language to refer to our study as a “randomized clinical trial” throughout.

Reviewer #2: PONE-D-20-11106: statistical review

SUMMARY. This is a two-step secondary analysis of a previous clinical trial of daily changes of step counts (primary outcome). Subjects are first segmented according to three groups, using latent class methods. Then, generalized mixed-effects models are estimated to compare each intervention arm to control within each latent class for the intervention and follow-up periods. I have very little to say about this paper. Latent class analysis is correct and generalized linear mixed-effects models appropriately account for latent heterogeneity and longitudinal correlation of the outcomes. I just list below some minor points that the authors should address to improve clarity and reproducibility of the results.

MINOR POINTS

1. Table 2 is obtained by exploiting the conditional probabilities of the observed variables given the latent class: the table of these probabilities should be included in the paper, perhaps as supplementary material.

This is now included as S1 Table in Supporting Information.

2. I guess that the mixed models that have been exploited are those of doi: 10.1001/jamainternmed.2019.3505. As a courtesy to the reader, please specify the kind of model you are using.

We specify that we used generalized mixed-effects models on page 8, line 170.

3. Please clarify that entropy (Table S1) is a measure of class separation and that, as such, it can be used for model selection.

The table (now Table S2) has been modified to include the footnote “Entropy is a measure of class separation with higher values indicating better separation.”

4. Although data are available without restriction, they are not attached. Data should be included as supplementary material and metadata provided.

The full dataset for our analyses has been included in Supporting Information as S3 Dataset.

Reviewer #3: The aim of this secondary analysis of a remotely delivered randomized clinical physical activity promotion trial was to identify behavioral phenotypes and compare physical activity changes for each intervention arm relative to control within each behavioral phenotype. This study uniquely fills a gap in the physical activity promotion literature, helping advance our understanding of which gamification interventions – a promising and increasingly popular behavior change approach – may be most effective for different population subgroups. The identification and evaluation of behavioral phenotypes that are based on a combination of variables as opposed to a single variable is an insightful perspective rooted in a sound conceptual basis that has been successfully applied in other sectors. The large sample, consideration of variables relevant to physical activity that could be screened somewhat easily, and evaluation of physical activity change both at the end of the intervention and follow up are study strengths. The rationale for the analytic approach is well described, key limitations are highlighted, and reasonable interpretations of the findings are made. Some very minor concerns are listed below.

Minor Concerns

Introduction
• p. 3, line 60: Could you please clarify or provide a few examples in parentheses of what you mean by baseline participant traits to help differentiate from the variables you examined?

We are referring to demographic variables such as age, sex, race/ethnicity and common baseline measures of health status and motivation.

We have modified the text on page 3, line 60 as follows: Similarly, comparisons within studies suggest that baseline participant traits such as age, sex, race/ethnicity and health status are not reliable predictors of participant response

Methods
• Although it is mentioned in your main outcomes manuscript for the trial, could you please mention the wearable device that was used? Also, is it a wrist-worn device?

We have modified the text on page 5, lines 108-110 as follows: Step counts were tracked using a wrist-worn wearable device (Withings Activite Steel), which was mailed to all participants along with a replacement battery.

Discussion
• Your interpretations of your findings are logical. Nonetheless, are there any findings at all from previous physical activity promotion studies that could be discussed to help situate your findings in the broader literature – anything similar or different from your findings?

As we state in the introduction, previous studies on physical activity promotion have suggested that traditional sociodemographic variables are not reliable predictors of intervention responsiveness. We referenced a study by Boslaugh et al. that showed using a combination of demographic, health status and psychosocial variables maximally differentiated the physical activity level of a sample of U.S. adults [15]. We have expanded our discussion section to highlight physical activity interventions that incorporate social features similar to the different social incentives evaluated in our study. Interventions that leverage social networks have become more common with the use of technology and mobile platforms. Interestingly, our findings indicate that the type of social incentive used may be relevant for some participant subgroups but not others. Further work is needed to confirm our findings and evaluate specific strategies for targeting social incentives to participant subgroups.

• It may not be a good idea to speculate too much, but do you have any thoughts about what could be explored in the future to help those who may have a more competitive orientation sustain their physical activity improvements? Or perhaps it could just be noted as a future area of inquiry if space permits.

Sustaining physical activity after the intervention period when incentives are removed has been a noted challenge in health behavior research. Our findings for class 1 participants who responded best to the competitive arm and were the largest class overall suggest that ongoing incentives and innovative incentive structures may be necessary and warrant further research.

We have modified the text on page 15, lines 294-297 as follows: Sustaining behavior change is known to be challenging after incentives are withdrawn [41]. Ongoing incentive programs may be necessary to support many individuals, including those with high baseline motivation and self-control, and should be explored in future work.

• It may be worth mentioning that only daily step counts were examined – other outcomes may be of interest too and could be explored in future research (MVPA etc.).

We agree that other outcomes should be considered in physical activity interventions. Our study did not collect data on intensity of physical activity to assess MVPA. The main trial paper also evaluated the secondary outcome of proportion of participant-days that step goals were achieved. Our group also plans to do future work exploring sleep as another outcome of this intervention.
